# Clinical and Prognostic Implications of Roundabout 4 (*Robo4*) in Adult Patients with Acute Myeloid Leukemia

**DOI:** 10.1371/journal.pone.0119831

**Published:** 2015-03-20

**Authors:** Yin-Kai Chen, Hsin-An Hou, Jih-Luh Tang, Jie-Yang Jhuang, Yan-Jun Lai, Ming-Cheng Lee, Yuan-Yeh Kuo, Wen-Chien Chou, Chieh-Yu Liu, Chung-Wu Lin, Shih-Sung Chuang, Chien-Yuan Chen, Mei-Hsuan Tseng, Chi-Fei Huang, Ying-Chieh Chiang, Fen-Yu Lee, Ming-Chih Liu, Chia-Wen Liu, Ming Yao, Shang-Yi Huang, Bor-Sheng Ko, Szu-Chun Hsu, Shang-Ju Wu, Woei Tsay, Yao-Chang Chen, Hwei-Fang Tien

**Affiliations:** 1 Division of Hematology, Department of Internal Medicine, National Taiwan University Hospital, Taipei, Taiwan; 2 Department of Internal Medicine, National Taiwan University Hospital, Yun-Lin branch, Yun-Lin, Taiwan; 3 Graduate Institute of Clinical Medicine, College of Medicine, National Taiwan University, Taipei, Taiwan; 4 Department of Pathology, Far Eastern Memorial Hospital, New Taipei City, Taiwan; 5 Graduate Institute of Oncology, College of Medicine, National Taiwan University, Taipei, Taiwan; 6 Department of Laboratory Medicine, National Taiwan University Hospital, Taipei, Taiwan; 7 Biostatistics Consulting Laboratory, Department of Nursing, National Taipei University of Nursing and Health Sciences, Taipei, Taiwan; 8 Department of Pathology, National Taiwan University Hospital, Taipei, Taiwan; 9 Department of Pathology, Chi-Mei Medical Center, Tainan, Taiwan; German Red Cross Blood Service Frankfurt, GERMANY

## Abstract

**Background:**

Robo4 is involved in hematopoietic stem/progenitor cell homeostasis and essential for tumor angiogenesis. Expression of Robo4 was recently found in solid tumors and leukemia stem cells. However, the clinical implications of *Robo4* expression in patients with acute myeloid leukemia (AML) remain unclear.

**Methods:**

We investigated the clinical and prognostic relevance of mRNA expression of *Robo4* in bone marrow (BM) mononuclear cells from 218 adult patients with *de novo* AML. We also performed immunohistochemical staining to assess the Robo4 protein expression in the BM biopsy specimens from 30 selected AML patients in the cohort.

**Results:**

Higher *Robo4* expression was closely associated with lower white blood cell counts, expression of HLA-DR, CD13, CD34 and CD56 on leukemia cells, t(8;21) and ASXL1 mutation, but negatively correlated with t(15;17) and *CEBPA* mutation. Compared to patients with lower *Robo4* expression, those with higher expression had significantly shorter disease-free survival (DFS) and overall survival (OS). This result was confirmed in an independent validation cohort. Furthermore, multivariate analyses showed that higher *Robo4* expression was an independent poor prognostic factor for DFS and OS in total cohort and patients with intermediate-risk cytogenetics, irrespective of age, WBC count, karyotype, and mutation status of *NPM1/FLT3-ITD*, and *CEBPA*.

**Conclusions:**

BM *Robo4* expression can serve as a new biomarker to predict clinical outcomes in AML patients and Robo4 may serve as a potential therapeutic target in patients with higher *Robo4* expression.

## Introduction

The Roundabout (Robo) family proteins are highly conserved transmembrane cell adhesion molecules that are expressed predominantly in neuronal tissues and play a role in the development of the nervous system. [1, 2] Roundabout 4 (Robo4), the fourth member of Robo family, was first identified by Huminiecki et al in 2002. [3] It is distinct from the other family members in that it is expressed specifically in endothelial cells [4,5] and hematopoietic stem/progenitor cells (HSPCs). [6,7] Robo4 acts as a receptor for Slit 2 and modulates vascular endothelial growth factor A (VEGF)–VEGF receptor signaling. [8,9] The Slit-Robo signaling is essential for angiogenesis, [6,10] especially tumor angiogenesis. [11] obo4 expression was found to be significantly up-regulated in tumor endothelia compared to their normal counterparts. [12] In addition to angiogenesis, Slit2-Robo4 signaling pathway also plays an essential role in BM homing and mobilization of HSPCs. [7,13] Smith-Berdan et al demonstrated that Robo4, in cooperation with CXCR4, acted as an HSPC-specific adhesion receptor to localize HSPC to BM niches. [7]

Acute myeloid leukemia (AML) is a heterogeneous hematological malignancy regarding the pathogenesis, clinical features and treatment outcomes. In addition to intrinsic changes of leukemic cells including cytogenetic, genetic and epigenetic alterations, impaired hematopoietic microenvironment of the bone marrow (BM) may also contribute to the development of AML. [14] It has been shown that adhesion of AML cells to the BM stromal cells promotes their survival and proliferation. [15,16] Angiogenesis also plays an important role in AML. [17,18] Higher BM angiogenesis predicts a higher relapse rate and poorer prognosis in AML patients. [18,19]

Robo4 has been shown to be expressed on leukemic stem cells. [20] However, little is known about the expression of *Robo4* and its clinical implications in AML. In the present study, we investigated the BM *Robo4* expression by quantitative real-time polymerase chain reaction (RQ-PCR) in a cohort of 218 adults with *de novo* AML and correlated the results with clinical features, major molecular mutations and outcomes of the patients. To the best of our knowledge, this is the first report to address the prognostic implication of *Robo4* expression in AML patients. We found that higher BM *Robo4* expression was an independent unfavorable prognostic factor for overall survival (OS) in these patients, and the finding was also validated in an independent validation cohort.

## Materials and Methods

### Patients

The original cohort comprised 218 patients with newly diagnosed *de novo* AML at the National Taiwan University Hospital (NTUH) from April 1996 to December 2007 who had complete clinical data and enough cryopreserved BM samples for analysis. Twenty normal marrow donors were also enrolled for comparison. Diagnosis and classification of AML were made according to the French-American-British (FAB) Cooperative Group Criteria. Among the 218 patients, 148 (67.9%) received standard intensive chemotherapy. The 134 non-M3 patients received induction chemotherapy with idarubicin 12 mg/m^2^ per day on days 1–3 and cytarabine 100 mg/m^2^ per day on days 1–7 and then consolidation chemotherapy with 2–4 courses of high-dose cytarabine (2000 mg/m^2^ q12h days 1–4, total 8 doses), with or without an anthracycline (idarubicin or novatrone), after achieving complete remission (CR). [21,22] The 14 patients with acute promyelocytic leukemia (M3 subtype) received concurrent all-trans retinoic acid and chemotherapy (idarubicin 12 mg/m^2^ per day on days 1–2) as induction treatment. Consolidation chemotherapy with anthracycline-based regimen was given for 4 courses after CR was achieved. The validation cohort comprised 53 adult AML patients diagnosed at the NTUH from Jan 2008 to June 2009. (S4 Table) These patients were treated with the same regimens as those in the original cohort and were used to validate the prognostic effect of BM *Robo4* expression in AML. This study was approved by the Institutional Review Board of the NTUH; and written informed consent was obtained from all participants in accordance with the Declaration of Helsinki.

### Quantitative real-time polymerase chain reaction (RQ-PCR)

BM mononuclear cells from 271 patients, including the original and validation cohorts, before chemotherapy and 20 healthy transplantation donors were isolated and cryopreserved until use. Total RNA was extracted and reverse transcribed. The gene expression level was quantified utilizing TaqMan technology on the Applied Biosystems 7500/7500 Fast Real-Time PCR System as previously described. [23] Gene-specific primers and probe of Robo4 were available as TaqMan Gene Expression Assay (Assay ID, Hs00219408_m1*, Applied Biosystems). Each sample was tested at least twice independently. The amount of the target gene was normalized to that of the housekeeping gene *RPLP0*. [17, 24, 25] The copies of target gene were quantified only after successful amplification of the internal control, using the standard curves derived from cloned plasmids. All data were presented as log ratio of the target gene/*RPLP0*.

### Immunohistochemical staining (IHC) for Robo4 protein

We performed IHC to assess the Robo4 protein expression in the BM biopsy specimens from 30 selected AML patients in the original cohort, 17 having higher *Robo4* mRNA expression and another 13, lower *Robo4* mRNA expression. The tissue section was prepared from formalin-fixed, paraffin-embedded tissue. After deparaffinization and rehydration as previously described, [23] the slides were boiled in 0.01 mol/L citrate buffer (pH = 6.1) for 5 minutes to retrieve the antigens. The endogenous peroxidase activity was blocked by incubation with 3% H_2_O_2_ in methanol for 30 minutes. Then the sections were blocked with 10% normal goat serum (VECTASTAIN Elite ABC kit; Vector Laboratories). The primary antibody we used was rabbit polyclonal anti-human Robo4 (PAB12049, Abnova) at a 1:250 dilution. A negative, no-antibody control was included for each staining. The slides were incubated with a biotinylated secondary antibody (1:200 dilution; goat anti-rabbit IgG; VECTASTAIN Elite ABC kit; Vector Laboratories) for 60 minutes at room temperature and AB reagent was applied based on the manufacturer's instructions (VECTASTAIN Elite ABC kit; Vector Laboratories). The antigen detection was conducted by a color reaction with 3,3'-diaminobenzidine (DAB peroxidase substrate kit; catalog number SK-4100; Vector Laboratories). Finally, the sections were counterstained with hematoxylin and mounted. Robo4 expression was assessed by staining intensity and frequency of positively stained cells. A score of 1–5 was calculated for each specimen according to the addition of the score of staining intensity (0 = none, 1 = weak, 2 = strong) and the score of percentage of myeloid cells positively stained (1 = 0~10%, 2 = 10~50%, 3>50%) by the pathologists who were blind to the results of *Robo4* mRNA expression. [26–28] Erythroid cells were excluded from calculation.

### Cytogenetics

BM mononuclear cells were harvested directly or after 1–3 day of unstimulated culture as described previously. [29] Metaphase chromosomes were banded by trypsin-Giemsa technique and karyotyped according to the International System for Human Cytogenetic Nomenclature.

### Immunophenotype analysis

A panel of monoclonal antibodies to myeloid associated antigens, including CD13, CD33, CD11b, CD15, CD14, and CD41a, as well as lymphoid-associated antigens, including CD2, CD5, CD7, CD19, CD10, and CD20, and lineage nonspecific antigens HLA-DR, CD34, and CD56 were used to characterize the immunophenotypes of the leukemia cells as previously described. [21]

### Mutation analysis

Mutation analyses of 17 relevant molecular alterations, including Class I mutations, such as *FLT3/*ITD and *FLT3/*TKD, [30] *NRAS*, [31] *KRAS*, [31] *JAK2*, [31] *KIT* [32] and *PTPN11* [33] mutations and Class II mutations, such as *MLL/*PTD, [34] *CEBPA* [35] and *RUNX1* [36] mutations, as well as mutations in *NPM1*, [37] *WT1*, [38] and those genes related to epigenetic modifications, such as *ASXL1*, [39] *IDH1*, [40] *IDH2*, [41] *TET2* [42] and *DNMT3A*, [21] were performed as previously described. Abnormal sequencing results were confirmed by at least two repeated analyses.

### Statistical analysis

Cut-off value of *Robo4* expression level was determined by sequential cutting strategy and was validated by bootstrap resampling method. The discrete variables of patients with lower and higher *Robo4* expression were compared using the Chi-square tests or Fisher’s exact test. We used Mann–Whitney *U*-test to compare continuous variables and medians of distributions. Whole patient population was included for analyses of the correlation between *Robo4* expression and clinical characteristics, however, only those receiving conventional standard chemotherapy, as mentioned above, were included in analyses of survivals. OS was measured from the date of first diagnosis to death from any cause or the last follow-up, whereas relapse was defined as a reappearance of at least 5% leukemic blasts in a BM aspirate or new extramedullary leukemia in patients with a previously documented CR. Disease-free status indicated that the patient achieved CR and did not relapse by the end of this study, and disease-free survival (DFS) was defined as the time from recruitment to the first of three events: treatment failure, leukemia relapse or death from any cause. To exclude confounding influences of different treatment regimens, patients who received allogeneic HSCT were censored on the day of cell infusion. [21, 22] We adopted Kaplan–Meier estimation to plot survival curves, and used log-rank tests to examine the difference between groups. Relative risk (RR) and 95% confidence interval (CI) were estimated by Cox proportional hazards regression models to determine independent risk factors associated with survival in multivariate analyses. Two-sided P-values less than 0.05 were considered statistically significant. All statistical analyses were accomplished with the SPSS 17 (SPSS Inc., Chicago, IL, USA) and Statsdirect (Cheshire, England, UK).

## Results

### Correlation of BM *Robo4* expression with clinical features and laboratory data in the original cohort

*Robo4* expression was significantly higher in AML patients than in normal BM donors. (P = 0.0007, S1 Fig.) The patients were then divided into two groups: one with lower expression of *Robo4* (n = 119) and the other with higher expression (n = 99), by using a cut-off point of 0.010 (*Robo4/RPLP0)*. The comparison of clinical characteristics between patients with lower and higher BM *Robo4* expression is shown in Table 1. Patients with higher BM *Robo4* expression had lower white blood cell (WBC) and blast counts than those with lower expression. There was no difference in sex, age and initial hemoglobin levels, platelet counts and serum lactate dehydrogenase levels between the two groups of patients. Compared to patients with lower BM *Robo4* expression, those with higher expression had less frequently FAB M3 subtype (P = 0.006, Table 1).

**Table 1:** Clinical manifestations of AML patients with higher and lower BM *Robo4* expression.

Variables	Total (n = 218)	Higher *Robo4* Expression (n = 99, 45.4%)	Lower *Robo4* Expression (n = 119, 54.6%)	P value
**Sex** ^1^				0.3373
Male	123	52 (42.3)	71 (57.8)	
Female	95	47 (49.5)	48 (50.5)	
**Age (year)** ^2^	54.5 (15–90)	55 (15–89)	54 (15–90)	0.4894
**Lab data** ^2^				
WBC (/μL)	23275 (380–423000)	17040 (650–175900)	27440 (380–423000)	0.0201
Hb (g/dL)	7.9 (3.3–16.2)	7.5 (3.3–13.6)	8.2 (3.5–16.2)	0.291
Platelet (×1,000 /μL)	40.5 (2–455)	46 (6–455)	37 (2–277)	0.2459
Blast (/μL)	10590 (0–369070)	7732 (0–141879)	14247 (0–369070)	0.0459
LDH (U/L)	857 (271–13130)	838 (298–5559)	917 (271–13130)	0.1824
**WHO Classification** ^1^				
**AML with recurrent genetic abnormalities**	121	51 (42.1)	70 (57.9)	0.3381
AML with t(8;21)(q22;q22),RUNX1-RUNX1T1	17	15 (88.2)	2 (11.8)	0.0002
AML with inv(16)(p13q22) or t(16;16)(p13;q22), CEFB-MYH11	9	6 (66.7)	3 (33.3)	0.3057
Acute promyelocytic leukemia with t(15;17)(q22;q12), PML-RARA	15	1 (6.7)	14 (93.3)	0.002
AML with t(9;11)(p22;q23);MLLT3-MLL	2	0	2 (100)	0.5019
AML with t(6;9)(p23;q34); DEK-NUP214	1	0	1 (100)	>0.9999
AML with inv(3)(q21q26.2) or t(3;3)(q21;q26.2); RPN1-EVI1	0	0	0	-
AML (megakaryoblastic) with t(1;22)(p13;q13); RBM15-MKL1	0	0	0	-
AML with mutated *NPM1*	53	23 (43.4)	30 (56.6)	0.7538
AML with mutated *CEBPA*	24	6 (25)	18 (75)	0.0488
**AML, not otherwise specified**	91	44 (48.4)	47 (51.6)	0.4922
AML with minimal differentiation (M0)	4	2 (50)	2 (50)	>0.9999
AML without maturation (M1)	25	13 (52)	12 (48)	0.5265
AML with maturation (M2)	23	10 (43.5)	13 (56.5)	>0.9999
Acute myelomonocytic leukemia (M4)	30	15 (50.0)	15 (50.0)	0.6937
Acute monoblastic/ acute monocytic leukemia (M5A/B)	7	2 (28.6)	5 (71.4)	0.4596
Acute erythroid leukemia (M6)	2	2 (100)	0	0.2051
Acute megakaryoblastic leukemia (M7)	0	0	0	-
Undetermined	6	4 (66.7)	2 (33.3)	0.4142
**Induction response** ^1^ ^,^ ^**3**^	148	61	87	
CR	107 (72.3)	39 (63.9)	68 (78.2)	0.0643
PR	4 (2.7)	2 (3.3)	2 (2.3)	0.3035
Refractory	26 (17.6)	13 (21.3)	13 (14.9)	0.3817
Induction death	11 (7.4)	7 (11.5)	4 (4.6)	0.2009
**Relapse**	50 (33.8)	20 (32.8)	30 (34.5)	0.5477

^1^number of patients (%)

^2^median (range)

^3^Only the 148 patients who received intensive chemotherapy were included.

Abbreviations: WHO, World Health Examination; CR, complete remission; PR, partial remission

Higher BM *Robo4* expression were positively associated with the expression of HLA-DR (P<0.0001), CD13 (P = 0.0417), CD34 (P = 0.0009) and CD56 (P = 0.039) on the leukemia cells (S1 Table). There was no difference in the expression of other antigens between the patients with higher and lower *Robo4* expression.

### Correlation between *Robo4* RNA expression and protein expression in the original cohort

Robo4 protein expression measured by scoring IHC of BM biopsy specimens correlated with *Robo4* mRNA expression in the 30 patients studied. The patients with higher scores measured by IHC had also higher *Robo4* mRNA expression than those with lower scores (P = 0.018). Representative IHC of a sample with a high score and the other one with a low score are demonstrated in S2 Fig. In addition to vasculoendothelial cells, Robo4 protein staining could be clearly seen in leukemia cells in the BM with a high score. Because not every patient in this study received BM biopsy, we only did IHC in selected patients. The correlation between Robo4 expression and angiogenesis in the BM of AML patients could not be analyzed in these limited samples.

### Correlation of BM *Robo4* expression with karyotypes and molecular gene mutations in the original cohort

Chromosome data were available in 207 (95%) patients at diagnosis. The comparison of cytogenetic changes between patients with higher and lower BM *Robo4* expression is shown in S2 Table. Higher *Robo4* expression was closely associated with chromosomal abnormality t(8;21), but inversely correlated with t(15;17) (15.8% *versus* 1.8%, P = 0.0002 and 1.1% *versus* 12.5%, P = 0.0019, respectively). With regard to the AML associated genetic alterations (Table 2), higher BM *Robo4* expression was closely associated with *ASXL1* mutation (15.2% *versus* 6.7%, P = 0.0489) but negatively associated with *CEBPA* mutation (6.1% *versus* 18.5%, P = 0.0076). AML patients with higher *Robo4* expression had a trend of higher probability of concurrent *DNMT3A* mutation and *KIT* mutation (P = 0.0707 and 0.0825, respectively).

**Table 2:** Association of BM ***Robo4*** expression level with other genetic alterations.

Variables	No. of patients with other genetic alteration (%)			P
	Whole cohort (n = 218)	Higher *Robo4* Expression (n = 99)	Lower *Robo4* Expression (n = 119)	
*FLT3/*ITD	54 (24.7)	20 (20.2)	34 (28.6)	0.1606
*FLT3/*TKD	17 (7.8)	4 (4.0)	13 (10.9)	0.0761
*NRAS*	26 (11.9)	14 (14.1)	12 (10.1)	0.4048
*KRAS*	7 (3.2)	4 (4.0)	3 (2.5)	0.7043
*PTPN11*	14 (6.4)	6 (6.1)	8 (6.7)	>0.9999
*KIT*	9 (4.1)	7 (7.1)	2 (1.7)	0.0825
*JAK2*	1 (0.5)	1 (1.0)	0	0.4541
*WTI*	12 (5.5)	6 (6.1)	6 (5.0)	0.7733
*NPM1*	53 (24.3)	23 (23.2)	30 (25.2)	0.7538
*CEBPA*	28 (12.8)	6 (6.1)	22 (18.5)	0.0076
*RUNX1*	29 (13.3)	14 (14.1)	15 (12.6)	0.8418
*MLL/*PTD	13 (6.0)	5 (5.1)	8 (6.7)	0.7758
*ASXL1*	23 (10.6)	15 (15.2)	8 (6.7)	0.0489
*IDH1*	13 (6.0)	5 (5.1)	8 (6.7)	0.7758
*IDH2*	31 (14.2)	18 (18.2)	13 (10.9)	0.1722
*TET2*	33 (15.1)	16 (16.2)	17 (14.3)	0.7089
*DNMT3A*	37 (17.0)	22 (22.2)	15 (12.6)	0.0707

### Impact of BM *Robo4* expression on clinical outcome in the original cohort

**Fig 1:**
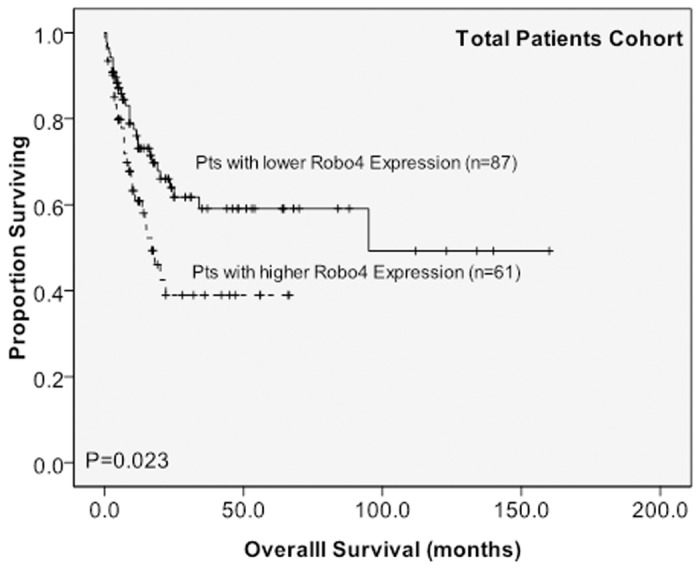
Kaplan–Meier survival curves for overall survival stratified by BM *Robo4* mRNA expression in the original cohort. Among a total of 148 patients with AML who received conventional intensive chemotherapy, patients with higher *Robo4* expression had shorter overall than those with lower *Robo4* expression.

**Fig 2:**
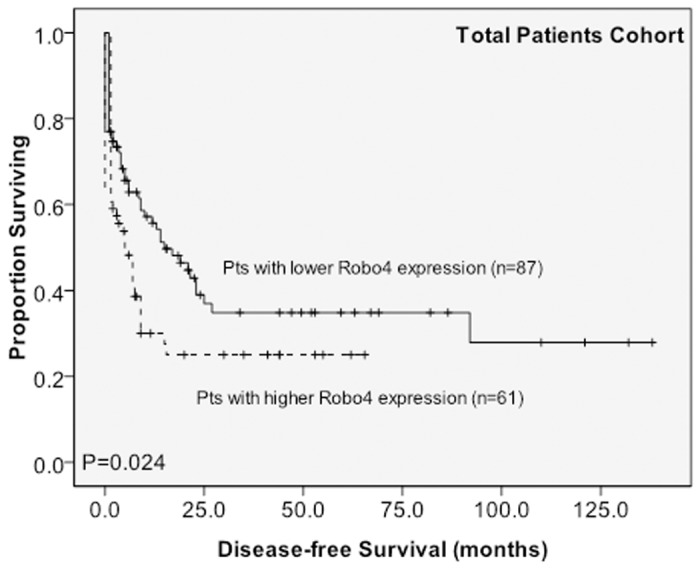
Kaplan–Meier survival curves for disease-free survival stratified by BM *Robo4* mRNA expression in the original cohort. Among a total of 148 patients with AML who received conventional intensive chemotherapy, patients with higher *Robo4* expression had shorter OS (A) and DFS (B) than those with lower *Robo4* expression.

Of the 148 AML patients receiving conventional intensive induction chemotherapy, 107 (72.3%) patients achieved a CR (Table 1). The patients with higher *Robo4* expression had a trend of a lower CR rate than those with lower *Robo4* expression, albeit the difference was not statistically significant (63.9% *versus* 78.2%, P = 0.0643). Similar trend was also found in the subgroup of 99 patients with intermediate-risk cytogenetics (CR rate, 59.5% *versus* 75.8%, P = 0.114) (S3 Table). With a median follow-up of 37.2 months (ranges, 1.0–160), patients with higher *Robo4* expression had significantly poorer OS (Fig. 1) and DFS (Fig. 2) than those with lower *Robo4* expression (median, 17.0 months *versus* 95.0 months, P = 0.023, and medium, 5.0 months *versus* 15.0 months, P = 0.024, respectively). In the subgroup of patients with intermediate-risk cytogenetics, the differences between the two groups in OS (S3 Fig.) (median, 13.5 months *versus* 95.0 months, P = 0.007) and DFS (S4 Fig.) (median, 4.0 months *versus* 10.0 months, P = 0.025) were even bigger.

In multivariate analysis (Table 3), the independent poor risk factors for OS were older age > 50 years, higher WBC count >50,000/μL, unfavorable karyotype, and higher *Robo4* expression (RR 1.779, 95% CI 1.005–3.149, P = 0.048). On the other hand, *CEBPA* ^double-mutation^ and *NPM1* ^+^/*FLT3-*ITD^-^ (mutant *NPM1* in the absence of *FLT3*-ITD) were independent favorable prognostic factors. The independent poor risk factors for DFS included older age > 50 years, higher WBC count >50,000/μL, unfavorable karyotype, and higher *Robo4* expression (RR 1.600, 95% CI 1.026–2.495, P = 0.038). *CEBPA* ^double-mutation^ and *NPM1* ^+^/*FLT3-*ITD^-^ were independent favourable factors for DFS.

**Table 3:** Multivariate Analysis (Cox regression) on the Disease-free Survival and Overall Survival.

Variables	Disease-free Survival					Overall Survival		
		95% CI				95% CI		
	RR	Lower	Upper	P value	RR	Lower	Upper	P value
Age^*1*^	1.98	1.286	3.048	0.002	3.497	1.982	6.17	0.001
WBC^*2*^	1.581	1.011	2.47	0.044	1.862	1.069	3.242	0.028
Karyotype^3^	4.175	2.634	6.62	<0.001	3.078	1.726	5.489	0.001
*NPM1/FLT3-*ITD^*4*^	0.246	0.11	0.554	0.001	0.398	0.166	0.955	0.039
*CEBPA* ^double mutation^	0.401	0.187	0.858	0.019	0.166	0.039	0.699	0.014
*Robo4* ^*5*^	1.6	1.026	2.495	0.038	1.779	1.005	3.149	0.048

Abbreviation: RR, relative risk; CI, confidence interval

^1^ Age > 50 relative to Age ≤50 (the reference)

^2^WBC greater than 50,000/μL *versus* less than 50,000/μL

^3^unfavorable cytogenetics *versus* others

^*4*^ *NPM1* ^+^ */FLT3-*ITD^-^ *versus* other subtypes

^*5*^Higher *Robo4* expression *versus l*ower *Robo4* expression

### The prognostic impact of BM *Robo4* expression in the validation cohort

**Fig 3:**
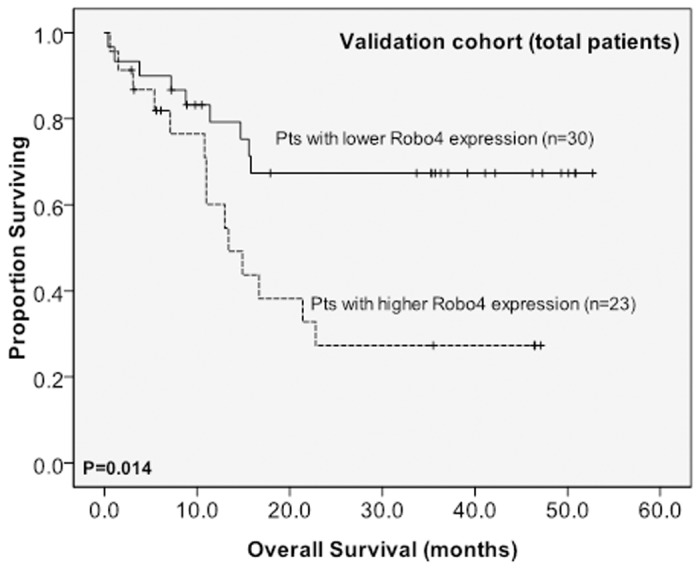
Kaplan-Meier survival curves for overall survival stratified by BM *Robo4* mRNA expression in the validation cohort. The patients with higher BM *Robo4* expression had a shorter OS than those with lower expression both among a total of 53 patients. The median value of BM *Robo4* expression in the original cohort of 148 patients was used as the cutoff point to define lower- and higher-expression groups.

**Fig 4:**
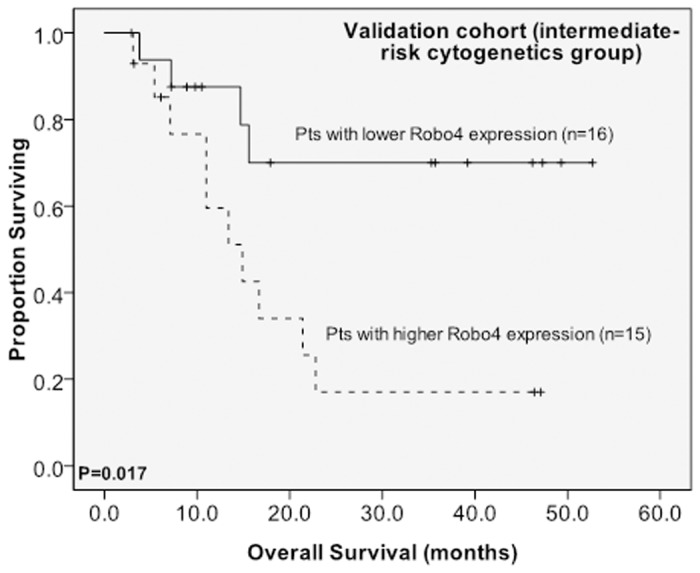
Kaplan-Meier survival curves for overall survival stratified by BM *Robo4* mRNA expression in the validation cohort. The patients with higher BM *Robo4* expression had a shorter OS than those with lower expression both among 25 patients with intermediate-risk cytogenetics. The median value of BM *Robo4* expression in the original cohort of 148 patients was used as the cutoff point to define lower- and higher-expression groups.

The cut-off point in the original cohort was used to define lower- and higher-expression groups in the validation cohort (n = 53). After a median follow-up time of 39.2 months (range 0.7 to 51.3), higher BM *Robo4* expression remained to be a significant unfavorable prognostic factor for OS in total patients (Fig. 3) and those with intermediate-risk cytogenetics in the validation cohort (Fig. 4) (median 13.4 months *versus* not reached, P = 0.014; median 14.2 months *versus* not reached, P = 0.017, respectively).

## Discussion

Robo4, a receptor for Slit2, is highly expressed in solid tumors, including brain, lung, urinary bladder and colorectal cancers and its expression is restricted to tumor vasculature but down-regulated in mature vasculature. [3, 43, 44] Interestingly, up-regulation of Robo4 was noticed in cancerous tissues but not in corresponding noncancerous part using paired samples in 50 patients with colorectal cancers. [43] Robo4 is thus an excellent tumor endothelial markers and a potential target for anti-angiogenesis therapy. [11,45] There have been few studies concerning the clinical relevance of Robo4 expression in cancers. Gorn et al demonstrated that elevated serum levels of Robo4 before treatment predicted poor prognosis in patients with non-small cell lung cancer, but it did not reach statistical significance in a univariate Cox regression model. [44] To the best of our knowledge, the current study was the first to evaluate *Robo4* expression and its clinical implications in AML patients. We demonstrated that *Robo4* expression was significantly elevated in AML patients compared to normal controls. Furthermore, higher BM *Robo4* expression predicted poorer DFS and OS in AML patients, especially those with intermediate-risk cytogenetics. The prognostic impact of higher BM *Robo4* expression was further validated in an independent validation cohort.

Robo4, in cooperation with CXCR4, plays a role in BM homing and mobilization of HSPCs. [7,13] It acts as an HSPC-specific adhesion receptor to localize HSPC to BM niches and its expression is dramatically down-regulated in mobilized HSPCs. [7] It has been shown that mobilization of AML cells by CXCR4 antagonist could enhance chemosensitivity of AML, which is translated into significant survival advantage. [46] Compatible with this finding, AML patients with lower *Robo4* expression in the present study were more sensitive to induction chemotherapy and had a trend of higher CR rates.

In the comprehensive analyses of the 17 genetic alterations and cytogenetic changes in the original cohort, we found that higher BM *Robo4* expression was closely associated with *ASXL1* mutation and t(8;21), but negatively associated with *CEBPA* mutation and t(15;17). The translocation t(8;21) is a frequent cytogenetic abnormality in AML; however, the presence of the resultant RUNX1-RUNXT1 fusion protein alone is not sufficient *per se* to induce leukemia. [47] In the present study, 15 of the 17 patients with t(8;21) showed higher *Robo4* expression (S2 Table). The higher *Robo4* expression, which may lead to dysregulation of angiogenesis or microenvironment, may be another hit in patients with this chromosomal abnormality. The significance of close association of higher *Robo4* expression and *ASXL1* mutation in the pathogenesis of AML needs further exploration.

## Conclusions

In conclusion, the present study provides evidences that higher BM *Robo4* expression is closely associated with distinct clinical and biologic characteristics in AML patients. Further, higher BM *Robo4* expression is an independent poor prognostic factor for OS. The unfavorable prognostic impact of higher BM *Robo4* expression was also validated in an independent validation cohort. Higher BM *Robo4* expression may serve as a new biomarker for foreseeing the clinical outcome of AML patients and may be a potential target for the treatment of AML patients with higher expression of this protein.

## Supporting Information

S1 Fig Comparison of BM *Robo4* expressions between AML patients in the original cohort and normal controls. The level was calculated as the log value of *Robo4* mRNA expression normalized to the housekeeping gene *RPLP0*. The P value was calculated using the Mann-Whitney *U* test.
(DOCX)

S2 Fig Representative immunohistochemical staining of Robo4 protein in BM biopsy specimens from two patients. One specimen from a patient with higher BM *Robo4* mRNA expression showed strong staining of leukemic cells (A & B) and another one from a patient with lower BM *Robo4* mRNA expression showed weak staining (C & D). (Magnification 100X and 400X, respectively)
(DOCX)

S3 Fig Kaplan–Meier survival curves for overall survival of AML patients with intermediate-risk cytogenetics stratified by BM *Robo4* mRNA expression in the original cohort. Among a total of 99 patients with intermediate-risk cytogenetics who received conventional intensive chemotherapy, patients with higher *Robo4* expression had shorter overall survival than those with lower expression.
(DOCX)

S4 Fig Kaplan–Meier survival curves for disease-free survival of AML patients with intermediate-risk cytogenetics stratified by BM *Robo4* mRNA expression in the original cohort. Among a total of 99 patients with intermediate-risk cytogenetics who received conventional intensive chemotherapy, patients with higher *Robo4* expression had shorter disease-free survival than those with lower expression.
(DOCX)

S1 Table Comparison of immune-phenotypes of leukemia cells between AML patients with higher and lower BM *Robo4* expression. (DOCX)

S2 Table Association of *Robo4* expression level with cytogenetic abnormalities*. (DOCX)

S3 Table Comparison of clinical manifestations between patients with higher and lower BM *Robo4* expression in AML patients with intermediate cytogenetics. (DOCX)

S4 Table Clinical characteristics of validation cohort. (DOCX)
