# Supplementary material for: Clinical and Prognostic Implications of Roundabout 4 (Robo4) in Adult Patients with Acute Myeloid Leukemia
Source: PLoS One. 2015 Mar 20;10(3):e0119831. doi: 10.1371/journal.pone.0119831 (PMC4368775; doi:10.1371/journal.pone.0119831)
Supplement: S1 Fig — The level was calculated as the log value of Robo4 mRNA expression normalized to the housekeeping gene RPLP0. The P value was calculated using the Mann-Whitney U test. (DOCX) [file pone.0119831.s001.docx]

**Figure S1.**

**P=0.0007**
