# Supplementary figures and images for: Clinical and Prognostic Implications of Roundabout 4 (Robo4) in Adult Patients with Acute Myeloid Leukemia
Source: PLoS One. 2015 Mar 20;10(3):e0119831. doi: 10.1371/journal.pone.0119831 (PMC4368775; doi:10.1371/journal.pone.0119831)

**Figure S2.**

**
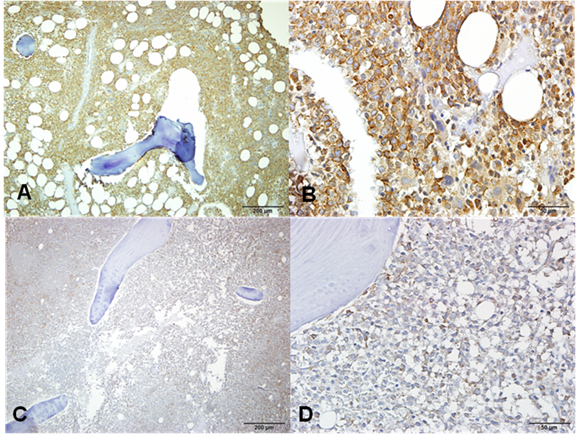
**

Supplement: S2 Fig — One specimen from a patient with higher BM Robo4 mRNA expression showed strong staining of leukemic cells (A & B) and another one from a patient with lower BM Robo4 mRNA expression showed weak staining (C & D). (Magnification 100X and 400X, respectively) (DOCX) [file pone.0119831.s002.docx]
