# Supplementary material for: Clinical and Prognostic Implications of Roundabout 4 (Robo4) in Adult Patients with Acute Myeloid Leukemia
Source: PLoS One. 2015 Mar 20;10(3):e0119831. doi: 10.1371/journal.pone.0119831 (PMC4368775; doi:10.1371/journal.pone.0119831)
Supplement: S3 Fig — Among a total of 99 patients with intermediate-risk cytogenetics who received conventional intensive chemotherapy, patients with higher Robo4 expression had shorter overall survival than those with lower expression. (DOCX) [file pone.0119831.s003.docx]

Figure S3A.
