# Supplementary material for: Clinical and Prognostic Implications of Roundabout 4 (Robo4) in Adult Patients with Acute Myeloid Leukemia
Source: PLoS One. 2015 Mar 20;10(3):e0119831. doi: 10.1371/journal.pone.0119831 (PMC4368775; doi:10.1371/journal.pone.0119831)
Supplement: S1 Table — (DOCX) [file pone.0119831.s005.docx]

**Table S1.**

**Comparison of immune-phenotypes of leukemia cells between AML patients with higher and lower BM *Robo4* expression**

| **Antigens** | **Total patients examined** | **Percentage of patients with the antigen expression** | | | **P** |
| --- | --- | --- | --- | --- | --- |
|  |  | **Whole cohort** | **Higher *Robo4* Expression** | **Lower *Robo4* Expression** |  |
| HLA-DR | 213 | 71.4 | 85.6 | 59.5 | <0.0001 |
| CD13 | 213 | 95.8 | 99.0 | 93.1 | 0.0417 |
| CD33 | 213 | 90.6 | 86.6 | 94.0 | 0.0971 |
| CD14 | 208 | 12.5 | 13.8 | 11.4 | 0.6755 |
| CD19 | 209 | 6.7 | 8.4 | 5.3 | 0.4133 |
| CD10 | 181 | 0.6 | 1.2 | 0 | 0.4696 |
| CD7 | 212 | 17.5 | 17.7 | 17.2 | >0.9999 |
| CD2 | 212 | 3.3 | 3.1 | 3.5 | >0.9999 |
| CD15 | 213 | 38.5 | 34.0 | 42.2 | 0.2585 |
| CD34 | 211 | 63.5 | 75.8 | 53.4 | 0.0009 |
| CD56 | 207 | 20.8 | 27.4 | 15.2 | 0.039 |
