# Supplementary material for: Clinical and Prognostic Implications of Roundabout 4 (Robo4) in Adult Patients with Acute Myeloid Leukemia
Source: PLoS One. 2015 Mar 20;10(3):e0119831. doi: 10.1371/journal.pone.0119831 (PMC4368775; doi:10.1371/journal.pone.0119831)
Supplement: S2 Table — (DOCX) [file pone.0119831.s006.docx]

**Table S2.**

**Association of *Robo4* expression level with cytogenetic abnormalities***

| **Variables** | **Total** | **Higher *Robo4* Expression** | **Lower *Robo4* Expression** | **P** |
| --- | --- | --- | --- | --- |
| **Karyotype^†,^**^‡^ **(N=207)** | |  |  | 0.116 |
| Favorable | 41 | 22 (23.2) | 19 (17.0) | 0.2964 |
| Intermediate | 145 | 60 (63.2) | 85 (75.9) | 0.0495 |
| Unfavorable | 21 | 13 (13.6) | 8 (7.1) | 0.1652 |
| **Cytogenetics**^‡^ |  |  |  | 0.166 |
| Normal | 109 | 45 (47.4) | 64 (57.1) |  |
| Abnormal | 98 | 50 (52.6) | 48 (42.9) |  |
| **Recurrent chromosomal abnormality**^‡^ | | | | |
| t(8;21) | 17 | 15 (15.8) | 2 (1.8) | 0.0002 |
| inv(16) | 9 | 6 (6.3) | 3 (2.5) | 0.4472 |
| t(15;17) | 15 | 1 (1.1) | 14 (12.5) | 0.0019 |

*Two hundred and seven patients, including 95 with higher *Robo4* expression and 112 with lower *Robo4* expression, had chromosome data at diagnosis.

^†^Favorable, t(15;17), t(8;21), inv (16) ; unfavorable, -7, del(7q), -5, del(5q), 3q abnormality, complex abnormalities; Intermediate, normal karyotype and other abnormalities.

^‡^number of patients (% of patients with the specific chromosomal change among the patients with higher or lower *Robo4* expression)
