# Supplementary material for: Clinical and Prognostic Implications of Roundabout 4 (Robo4) in Adult Patients with Acute Myeloid Leukemia
Source: PLoS One. 2015 Mar 20;10(3):e0119831. doi: 10.1371/journal.pone.0119831 (PMC4368775; doi:10.1371/journal.pone.0119831)
Supplement: S3 Table — (DOCX) [file pone.0119831.s007.docx]

**Table S3.**

**Comparison of clinical manifestations between patients with higher and lower BM *Robo4* expression in AML patients with intermediate cytogenetics**

| **Variables** | **Total**  **(n=99)** | **Higher *Robo4* Expression**  **(n=37, 37.4%)** | **Lower *Robo4***  **Expression**  **(n=62, 62.6%)** | | **P value** |
| --- | --- | --- | --- | --- | --- |
| **Sex**^†^ |  |  |  | | >0.999 |
| Male | 50 | 16 (43.2) | | 34 (54.8) |  |
| Female | 49 | 21 (56.8) | | 28 (45.2) |  |
| **Age (year)**^‡^ | 47 (15-78) | 55 (15-89) | | 44 (16-78) | 0.798 |
| **Lab data**^‡^ |  |  | |  |  |
| WBC (/μL) | 29500 (650-423000) | 29500 (650-160860) | | 30095 (2420-423000) | 0.076 |
| Hb (g/dL) | 7.9 (3.7-12.9) | 7.8 (3.7-11.4) | | 8.1 (4.2-12.9) | 0.361 |
| Platelet (×1,000 /μL) | 45.0 (6-331) | 46 (8-331) | | 44 (6-277) | 0.874 |
| Blast (/μL) | 19370 (0-338400) | 25603 (501-100100) | | 18992 (0-338400) | 0.019 |
| LDH (U/L) | 917 (286-13130) | 889 (298-2830) | | 917 (286-13130) | 0.950 |
| **FAB**^†^ |  |  | |  |  |
| M0 | 1 | 0 (0.0) | | 1 (100.0) | >0.999 |
| M1 | 30 | 9 (30.0) | | 21 (70.0) | 0.371 |
| M2 | 31 | 11 (46.3) | | 20 (64.5) | 0.827 |
| M3 | 0 | 0 | | 0 |  |
| M4 | 29 | 12 (41.4) | | 17 (58.6) | 0.651 |
| M5 | 7 | 5 (71.4) | | 2 (28.6) | 0.099 |
| M6 | 1 | 0 (0.0) | | 1 (100.0) | >0.999 |
| M7 | 0 | 0 | | 0 |  |
| **Induction response**^†^ | 99 | 37 | | 62 |  |
| CR | 69 (69.7) | 22 (59.5) | | 47 (75.8) | 0.114 |
| Non-CR | 23 (23.2) | 11 (29.7) | | 12 (19.4) | 0.325 |
| Induction death | 7 (7.1) | 4 (10.8) | | 3 (4.8) | 0.419 |
| **Median Overall Survival** |  |  | |  |  |
| **Allo-SCT** | 24 (24.2) | 10 (27.0) | | 14 (22.6) | 0.635 |

^†^number of patients (%)

^‡^median (range)

**^＊^** Patients who received intensive chemotherapy were included.

Abbreviations: FAB, French-American-British classification; CR, complete remission; PR, partial remission; Allo-SCT, allogeneic stem cell transplantation
