# Supplementary material for: Clinical and Prognostic Implications of Roundabout 4 (Robo4) in Adult Patients with Acute Myeloid Leukemia
Source: PLoS One. 2015 Mar 20;10(3):e0119831. doi: 10.1371/journal.pone.0119831 (PMC4368775; doi:10.1371/journal.pone.0119831)
Supplement: S4 Table — (DOCX) [file pone.0119831.s008.docx]

**Table S4**

**Clinical characteristics of validation cohort**

| **Variables** | **Total (n=53)** |
| --- | --- |
| **Sex^†^** |  |
| Male | 31 |
| Female | 22 |
| **Age (year)^‡^** | 45 (16-83) |
| Lab data^‡^ |  |
| WBC (/μL) | 23275 (490-246330) |
| Hb (g/dL) | 7.5 (3.7-13.1) |
| Platelet (×1,000 /μL) | 45 (5-366) |
| Blast (/μL) | 2688 (0-206276) |
| LDH (U/L) | 782 (325-5723) |
| **FAB^†^** |  |
| M0 | 3 (5.6) |
| M1 | 9 (17) |
| M2 | 18 (34) |
| M3 | 12 (22.6) |
| M4 | 8 (15.1) |
| M5 | 1 (1.9) |
| M6 | 2 (3.8) |
| **Induction response^†＊^** | 53 |
| CR | 42 (80.8) |
| Not CR | 11 (19.2) |

^†^number of patients (%)

^‡^median (range)

**^＊^** Patients who received intensive chemotherapy were included.

Abbreviations: FAB, French-American-British classification; CR, complete remission; PR, partial remission; Allo-SCT, allogeneic stem cell transplantation
